# Ceramide species are elevated in human breast cancer and are associated with less aggressiveness

**DOI:** 10.18632/oncotarget.24903

**Published:** 2018-04-13

**Authors:** Kazuki Moro, Tsutomu Kawaguchi, Junko Tsuchida, Emmanuel Gabriel, Qianya Qi, Li Yan, Toshifumi Wakai, Kazuaki Takabe, Masayuki Nagahashi

**Affiliations:** ^1^ Division of Digestive and General Surgery, Niigata University Graduate School of Medical and Dental Sciences, Niigata City 951-8510, Japan; ^2^ Breast Surgery, Department of Surgical Oncology, Roswell Park Comprehensive Cancer Center, Buffalo, New York 14263, USA; ^3^ Department of Biostatistics and Bioinformatics, Roswell Park Comprehensive Cancer Center, Buffalo, New York 14263, USA; ^4^ Department of Surgery, University at Buffalo Jacobs School of Medicine and Biomedical Sciences, The State University of New York, Buffalo, New York 14203, USA; ^5^ Department of Breast Surgery and Oncology, Tokyo Medical University, Tokyo 160-8402, Japan; ^6^ Department of Surgery, Yokohama City University, Yokohama 236-0004, Japan

**Keywords:** breast cancer, ceramide, metabolism, prognosis, sphingolipid

## Abstract

Sphingolipids have emerged as key regulatory molecules in cancer cell survival and death. Although important roles of sphingolipids in breast cancer progression have been reported in experimental models, their roles in human patients are yet to be revealed. The aim of this study was to investigate the ceramide levels and its biosynthesis pathways in human breast cancer patients. Breast cancer, peri-tumor and normal breast tissue samples were collected from surgical specimens from a series of 44 patients with breast cancer. The amount of sphingolipid metabolites in the tissue were determined by mass spectrometry. The Cancer Genome Atlas was used to analyze gene expression related to the sphingolipid metabolism. Ceramide levels were higher in breast cancer tissue compared to both normal and peri-tumor breast tissue. Substrates and enzymes that generate ceramide were significantly increased in all three ceramide biosynthesis pathways in cancer. Further, higher levels of ceramide in breast cancer were associated with less aggressive cancer biology presented by Ki-67 index and nuclear grade of the cancer. Interestingly, patients with higher gene expressions of enzymes in the three major ceramide synthesis pathways showed significantly worse prognosis. This is the first study to reveal the clinical relevance of ceramide metabolism in breast cancer patients. We demonstrated that ceramide levels in breast cancer tissue were significantly higher than those in normal tissue, with activation of the three ceramide biosynthesis pathways. We also identified that ceramide levels have a significant association with aggressive phenotype and its enzymes have prognostic impact on breast cancer patients.

## INTRODUCTION

Breast cancer is the leading cause of cancer-related deaths among women worldwide, and the estimated number of deaths is over 40,000 among women in the US in 2018 [1]. In order to improve mortality from breast cancer, it is crucial to understand its biology to develop innovative treatment strategies. There has been increasing evidence that lipid mediators such as ceramide [2] and sphingosine-1-phosphate (S1P) [3] play critical roles in several human cancers, including breast [4, 5], colon [6], lung [7], gastric [8, 9], and prostate cancer [10]. S1P is generated inside the cancer cells, and exported out of the cells where it regulates many functions in tumor microenvironment by binding to specific G protein-coupled receptors expressed either in cancer cells or host cells, known as the “inside-out” signaling of S1P [11]. In addition, we have demonstrated that S1P levels are high not only in tumors [4], but also in the tumor microenvironment [12]. We further demonstrated that high S1P production by tumor is associated with lymph node metastasis [13], indicating that S1P promotes cancer metastasis by affecting tumor microenvironment in human breast cancer. Therefore, it was of interest to analyze the ceramide levels of cancer, peri-tumor, normal breast tissue and interstitial fluid (that is a component of tumor microenvironment), since ceramide has not been as extensively studied and may have important relationships with cancer.

**Figure 1:**
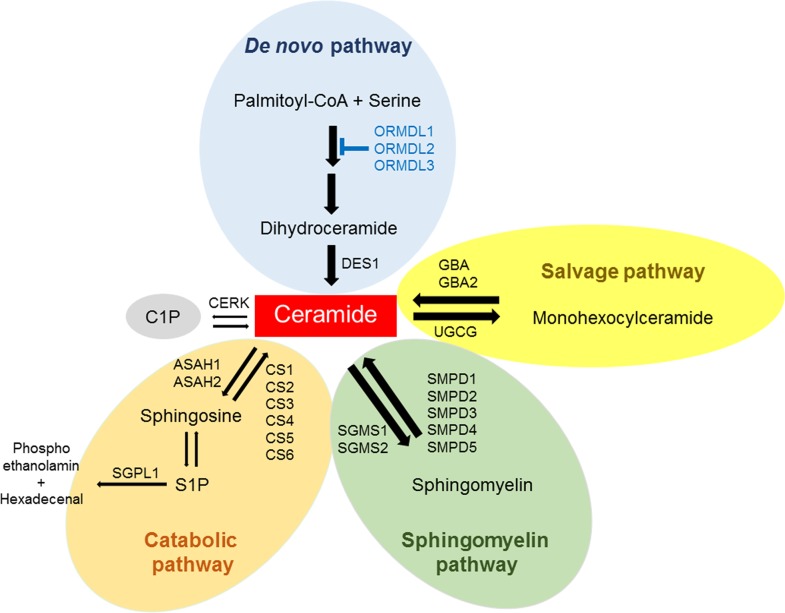
Pathways for ceramide metabolism in breast cancer tissue Ceramide is generated from the major pathways such as the *de novo* pathway, the salvage pathway and the sphingomyelin pathway. Ceramide can be phosphorylated to ceramide-1-phosphate (C1P), broken down to sphingosine and phosphorylated to sphingosine-1-phosphate (S1P). The gene expression of ceramide metabolism in The Cancer Genome Atlas (TCGA) cohort (n=112) were analyzed.

Ceramide is membrane lipid with important functions in regulating membrane fluidity and subdomain structures [14, 15]. Ceramide, a central component of the sphingolipid metabolic pathway [2], is generated via three separate but related pathways, including the salvage pathway, the sphingomyelin pathway, and the de novo pathway [16] (Figure 1). Ceramide can be generated in response to several stressors such as ionizing radiation, ultraviolet light, tumor necrosis factor (TNF-α), and chemotherapeutic agents [17]. In fact, ceramide is known to be a key regulator of chemotherapy-induced cell death from agents such as taxane [18]. Advances in sphingolipid research suggest that ceramides are the highly bioactive molecular species and have a great impact on cellular signaling and disease. Ceramide has been described to mediate cell death in cancer by inducing apoptosis [19], and its tumor suppressive functions are influenced by the nature of the stimulus, co-stimulatory signals, cell types, and enzymatic pathways [20]. It is well known that ceramides can activate specific kinases and protein phosphatases, and that these enzymes play key roles in the actions of ceramide [16, 21–24].

Recent advances in technology to determine the exact levels of sphingolipids not only *in vitro*, but also in *in vivo* tissue samples, provide an understanding of the role of ceramide in human breast cancer and its potential mechanisms [17]. Moreover, next generation sequencing-based comprehensive analysis and bioinformatics help to capture the broad picture of sphingolipid metabolism and enable researchers to calculate its impact on the survival of human patients [18]. In the setting of this background work, the aim of the current study was to determine the relevance of ceramide levels and its biosynthetic pathways in breast cancer patients, utilizing these advanced technologies. In this study, we demonstrated that ceramide levels in breast cancer tissue were significantly higher than those in normal breast tissue, with activation of the three ceramide biosynthesis pathways. We also identified that ceramide levels have a significant association with cell proliferation potency, and its enzymes have prognostic impact on breast cancer patients.

## RESULTS

### Patient characteristics

A total of 44 patients with primary breast cancer were included in the present study according to the above inclusion criteria. Clinicopathological characteristics are summarized in Table 1. Thirty-four (77.3%) patients were estrogen receptor (ER) positive, and 15 patients (34.1%) were HER2 positive. There was significant difference between the level of total ceramide in breast cancer tissues and hormone receptor status. In the present study, the total ceramide means refers to the sum of ceramides from C_14:0_ to C_26:1_, not including ceramide-1-phosphate (C1P). The total ceramide level was higher in hormone receptor positive breast cancer than in negative breast cancer. There was no significant difference in HER2 status. Interestingly, there was no significant difference in ceramide levels between patients with lymph node metastasis and those without. However, this finding could have been limited by the small number of cases with lymph node metastasis. Conversely, there was a significant difference in S1P levels as reported previously [13] (Table 2).

**Table 1:** Baseline characteristics of breast cancer cohort (n = 44)

Variables	Number	%
Age (years)		
< 60	23	52.3
≥ 60	21	47.7
BMI		
< 25	30	68.2
≥ 25	14	31.8
Pathologic T stage		
T1	18	40.9
T2	21	47.7
T3	5	11.4
Pathologic N stage		
N0	40	90.9
N1	4	9.1
Estrogen receptor status		
Negative	10	22.7
Positive	34	77.3
Progesterone receptor status		
Negative	16	36.4
Positive	28	63.6
HER2 status		
Negative	29	65.9
Positive	15	34.1
Ki-67 index		
<15%	23	52.3
15%-30%	12	27.2
>30%	9	20.5
Nuclear grade		
1	24	54.5
2	9	20.5
3	11	25

BMI, body mass index; HER2, human epidermal growth factor receptor 2.

**Table 2:** Ceramide levels of tissue samples and of the subgroups tumor, size and LN status are shown

	Number of patients	C_total_	p value	C_14:0_	p value	C_16:0_	p value	C_18:1_	p value	C_18:0_	P value
Tumor size											
≤ 2cm	18	3.799	**0.030**	0.051	**0.034**	1.152	0.197	0.086	0.377	0.140	0.159
>2cm	26	7.327		0.080		1.375		0.097		0.199	
LN status											
Negative	40	5.073	0.541	0.069	0.441	1.344	0.373	0.095	0.292	0.152	0.338
Positive	4	3.545		0.048		0.865		0.058		0.172	
ER status											
Negative	10	3.229	**0.026**	0.047	0.168	0.821	**0.041**	0.060	0.238	0.112	0.339
Positive	34	5.871		0.075		1.592		0.098		0.155	
PgR status											
Negative	16	3.602	**0.015**	0.042	**0.017**	1.035	0.081	0.064	0.366	0.169	0.577
Positive	28	5.982		0.087		1.679		0.096		0.152	
HER2 status											
Negative	29	6.158	0.055	0.086	**0.028**	1.557	0.271	0.134	0.340	0.152	0.393
Positive	15	3.602		0.053		1.079		0.071		0.119	

	C_20:0_	p value	C_22:0_	p value	C_24:1_	p value	C_24:0_	p value	C_26:1_	p value	C_26:0_	p value
Tumor size												
<2cm	0.158	0.073	0.437	**0.025**	0.924	**0.045**	0.651	**0.012**	0.485	**0.009**	0.008	**0.016**
≥ 2cm	0.213		1.036		2.386		1.400		0.119		0.021	
LN status												
Negative	0.206	0.207	0.579	0.395	1.248	0.373	0.840	0.829	0.079	0.595	0.011	0.239
Positive	0.137		0.417		0.877		0.774		0.095		0.026	
ER status												
Negative	0.117	**0.047**	0.221	**0.019**	0.847	0.051	0.402	**0.044**	0.048	0.168	0.007	0.186
Positive	0.207		0.798		1.435		0.885		0.092		0.013	
PgR status												
Negative	0.123	**0.028**	0.264	**0.011**	0.902	**0.014**	0.356	0.095	0.049	0.085	0.007	**0.015**
Positive	0.219		0.845		1.820		0.973		0.097		0.014	
HER2 status												
Negative	0.208	0.177	0.782	0.116	1.522	0.155	0.097	0.105	0.097	0.055	0.012	0.379
Positive	0.151		0.510		0.911		0.563		0.048		0.011	

HER2, human epidermal growth factor receptor 2.

### Ceramide levels are significantly higher in breast cancer than the paired normal breast tissue

**Figure 2:**
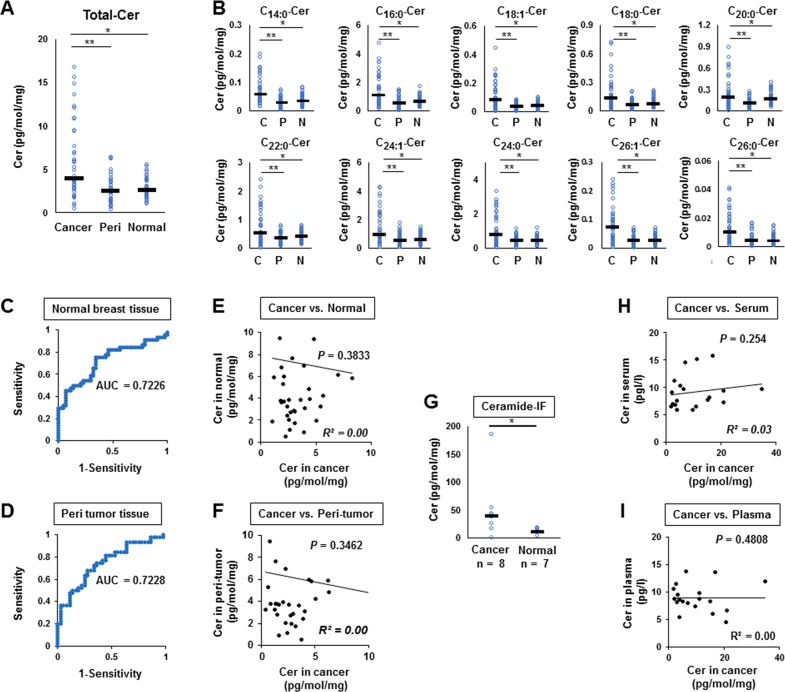
Ceramide levels in breast cancer tissue (n = 44), peri-tumor tissue (n = 36), and normal breast tissue (n = 44) **(A, B)**, Levels of total ceramide (Cer) (A) and each ceramide specie (C14:0, C16:0, C18:1, C18:0, C20:0, C22:0, C24:1, C24:0, C26:1 and C26:0) (B) in cancer tissue (Cancer or C), peri-tumor tissue (Peri or P) and normal tissue (Normal or N) were determined by mass spectrometry. Mean values are shown by the horizontal lines. ^*^, *P*<0.05 for cancer vs. normal tissue; ^**^, *P*<0.05 for cancer vs. peri-tumor tissue. **(C, D)**, Receiver-operating characteristic (ROC) curves and the area under the ROC curve (AUC) were produced to assess the ability of our ceramide assays to distinguish cancer tissue from either normal breast tissue sample (C) or peri-tumor tissue (D). **(E, F)**, Correlation between the ceramide level in breast cancer tissue and that in normal breast tissue (E) or peri-tumor (F) were compared in individual patient. The correlation between two variables is denoted by R^2^. **(G)** The ceramide levels in interstitial fluid (IF) of cancer tissue and normal breast tissue were determined. **(H, I)** Correlation between the ceramide level in breast cancer tissue and that in serum (H) or plasma (I) were compared. The correlation between two variables is denoted by R^2^.

We measured the ceramide levels in breast cancer (n=44), peri-tumor (n=36), and normal breast tissues (n=44) (Figure 2). The levels of total ceramide in cancer tissue were significantly higher compared to normal breast tissue (n=44, *p*<0.0001) or peri-tumor tissues (n=36, *p*<0.0001) (Figure 2A). Elevation of levels in cancer compared to peri-tumor or normal breast tissue were consistent throughout all ceramide species (C14:0, C16:0, C18:1, C18:0, C20:0, C22:0, C24:1, C24:0, C26:1 and C26:0) (Figure 2B).

Receiver-operating characteristic (ROC) curves and the area under the ROC curve (AUC) were used to assess the prognostic ability of ceramide levels to distinguish cancer tissue from either normal breast tissue (Figure 2C) or peri-tumor tissue (Figure 2D). The AUC scores were 0.7226 for normal tissue (Figure 2C) and 0.7228 for peri-tumor tissue (Figure 2D), respectively, showing that breast cancer tissue may be distinguished from normal breast tissue by ceramide levels. We analyzed the correlation between the ceramide level of breast cancer tissue and that of normal breast tissue (Figure 2E), or peri-tumor in individual patients (Figure 2F), and found no correlation between them (R^2^=0.00, p=0.38, and R^2^ =0.00, p=0.35, respectively). The ceramide levels in interstitial fluid (IF) of cancer tissue were significantly higher than those of normal breast tissue (*p*=0.018) (Figure 2G). On the other hand, the serum and plasma ceramide levels of breast cancer patients, of which 19 patients were available for analysis, were not associated with ceramide level in cancer tissue of each patient (Figure 2H, 2I). These results suggest that ceramides are present in tumor microenvironment, but do not leak out from the tumor into the systemic circulation.

### The three major pathways of ceramide biosynthesis are activated to increase ceramide in cancer

**Figure 3:**
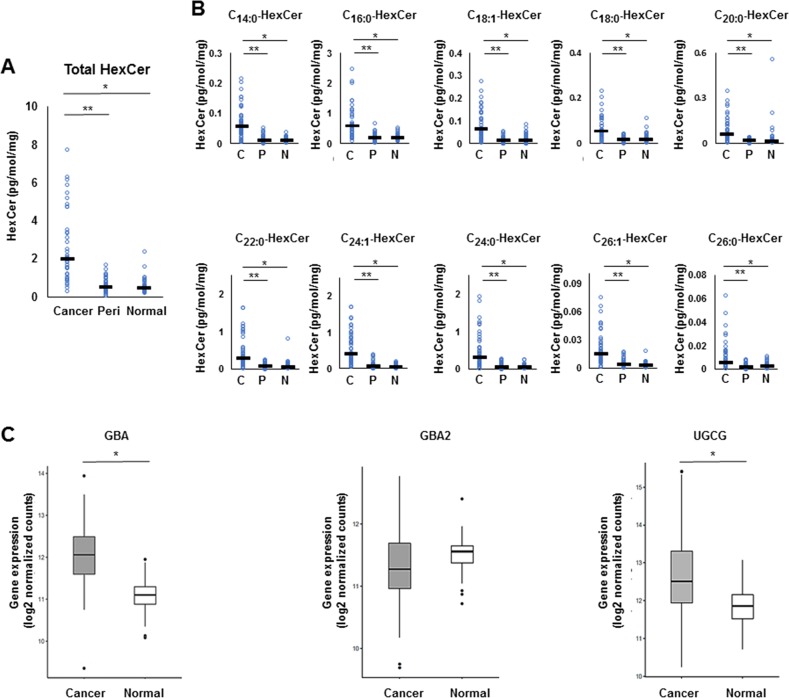
The metabolism in the salvage pathway for ceramide synthesis in breast cancer tissue **(A, B)** Levels of total monohexocylceramide (HexCer) (A) and each monohexocylceramide specie (C14:0, C16:0, C18:1, C18:0, C20:0, C22:0, C24:1, C24:0, C26:1 and C26:0) (B) in cancer tissue (Cancer or C), peri-tumor tissue (Peri or P) and normal tissue (Normal or N) were determined by mass spectrometry. Mean values are shown by the horizontal lines. ^*^, *P*<0.05 for cancer vs. normal tissue; ^**^, *P*<0.05 for cancer vs. peri-tumor tissue. **(C)** The expression of glucosylceramidase (GBA) and uridine diphosphoglucose ceramide glucosyltransferase (UGCG) in The Cancer Genome Atlas (TCGA) cohort (n=112) were analyzed. Mean values are shown by the horizontal lines. ^*^, P<0.05 for cancer vs. normal tissue.

We investigated the salvage pathway, one of the three major pathways of ceramide biosynthesis, as a source of ceramide in human breast cancer tissues (Figure 1). Levels of monohexocylceramide, which is the core molecule of many glycosphingolipids involved in the salvage pathway, were measured in the breast cancer patient cohort. Total monohexocylceramide levels as well as levels of all the monohexocylceramide species were significantly higher in cancer tissue than that in peri-tumor or in normal breast tissue (Figure 3A, 3B). These results suggest that all of the monohexocylceramide species as well as the total amount of monohexocylceramides were significantly higher in cancer.

To evaluate the expression levels of ceramide-producing enzymes in the salvage pathway, gene expression of glucosylceramidase (GBA), encoding a lysosomal membrane protein that generates ceramide, was analyzed in the TCGA cohort [16]. The expression levels of GBA were significantly higher in breast cancer tissue than in paired normal breast tissue (TCGA cohort, n = 112) The expression levels of uridine diphosphoglucose ceramide glucosyltransferase (UGCG) were also significantly higher in breast cancer tissue than in paired normal breast tissue (Figure 3C).

**Figure 4:**
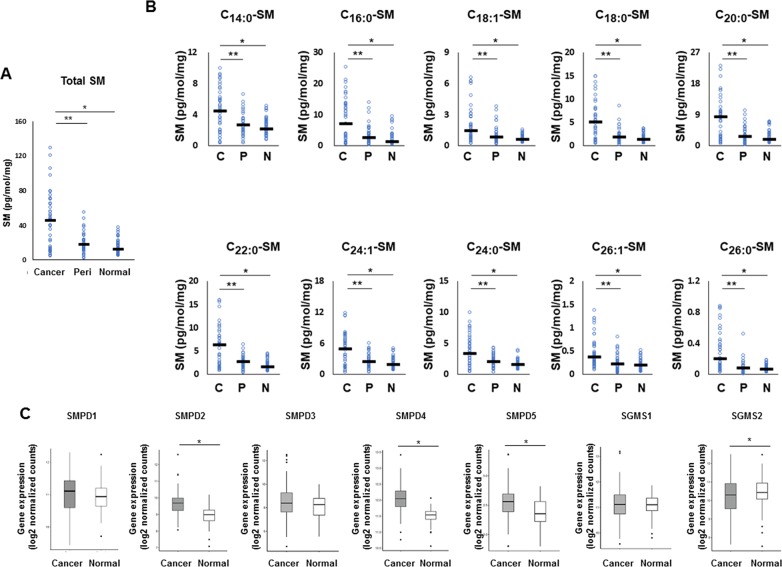
The metabolism in the sphingomyelin pathway for ceramide synthesis in breast cancer patients **(A, B)** Levels of total sphingomyelin (SM) (A) and each sphingomyelin species (C14:0, C16:0, C18:1, C18:0, C20:0, C22:0, C24:1, C24:0, C26:1 and C26:0) (B) in cancer tissue (Cancer or C), peri-tumor tissue (Peri or P) and normal tissue (Normal or N) were determined by mass spectrometry. Mean values are shown by the horizontal lines. ^*^, *P*<0.05 for cancer vs. normal tissue; ^**^, *P*<0.05 for cancer vs. peri-tumor tissue. **(C)** The expression of sphingomyelin phosphodiesterase 2 (SMPD2), sphingomyelin phosphodiesterase 4 (SMPD4), sphingomyelin phosphodiesterase 5 (SMPD5) and sphingomyelin synthase 2 (SGMS2) in The Cancer Genome Atlas (TCGA) cohort (n=112) were analyzed. Mean values are shown by the horizontal lines. ^*^, P<0.05 for cancer vs. normal tissue.

We next investigated the sphingomyelin pathway, another major pathway of ceramide biosynthesis. All sphingomyelin species were significantly higher in cancer tissue than that in peri-tumor or normal breast tissue, as well as the total levels of sphingomyelin determined by mass spectrometry (Figure 4A, 4B). In order to examine whether the sphingomyelin pathway is involved in increased production of ceramides in cancer, expression of three genes that code enzymes related to ceramide metabolism were analyzed. These included sphingomyelin phosphodiesterase 2 (SMPD2), sphingomyelin phosphodiesterase 4 (SMPD4), and sphingomyelin phosphodiesterase 5 (SMPD5). These enzymes regulate sphingomyelinase to increase ceramide biosynthesis as well as sphingomyelin synthase 2 (SGMS2), which regulates sphingomyelin phosphodiesterase to increase conversion of ceramide to sphingomyelin [21]. We found that SMPD2, SMPD4, and SMPD5 were significantly higher in breast cancer tissue than that in paired normal breast tissue, while SGMS2 was significantly decreased in breast cancer tissue (TCGA cohort, n=112) (Figure 4C).

**Figure 5:**
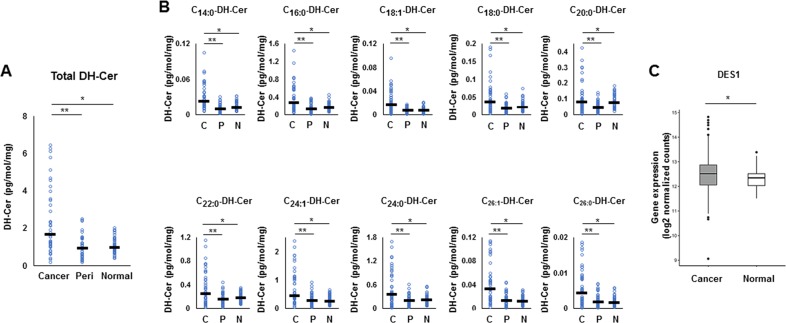
The metabolism in the *de novo* pathway for ceramide synthesis in breast cancer patients **(A, B)** Levels of total dihydroceramide (DH-Cer) (A) and each dihydroceramide species (C14:0, C16:0, C18:1, C18:0, C20:0, C22:0, C24:1, C24:0, C26:1 and C26:0) (B) in cancer tissue (Cancer or C), peri-tumor tissue (Peri or P) and normal tissue (Normal or N) were determined by mass spectrometry. Mean values are shown by the horizontal lines. ^*^, *P*<0.05 for cancer vs. normal tissue; ^**^, *P*<0.05 for cancer vs. peri-tumor tissue. **(C)** The expression of Dihydroceramide desaturase 1 (DES1) in The Cancer Genome Atlas (TCGA) cohort (n=112) were analyzed. Mean values are shown by the horizontal lines. ^*^, *P*<0.05 for cancer vs. normal tissue.

As for the *de novo* pathway, the levels of all dihydroceramide species, which serve as the precursor of ceramide in the *de novo* pathway, were significantly higher in cancer tissue than that in peri-tumor or normal breast tissue (Figure 5A, 5B). In order to determine whether the *de novo* pathway contributes to the increase of ceramides in cancer, gene expression of dihydroceramide desaturase 1 (DES-1), an enzyme that converts dihydroceramide to ceramide in the *de novo* pathway, was measured. DES1 expression was significantly higher in breast cancer tissue than that in paired normal breast tissue (TCGA cohort, n=112) (Figure 5C). Taken together, these findings suggest that both the amount of the substrates and the expression of the ceramide-producing enzymes were increased in all of the three major ceramide synthesis pathways in breast cancer compared to normal breast tissue.

### The other sphingolipid metabolic pathways are also activated to increase ceramide

**Figure 6:**
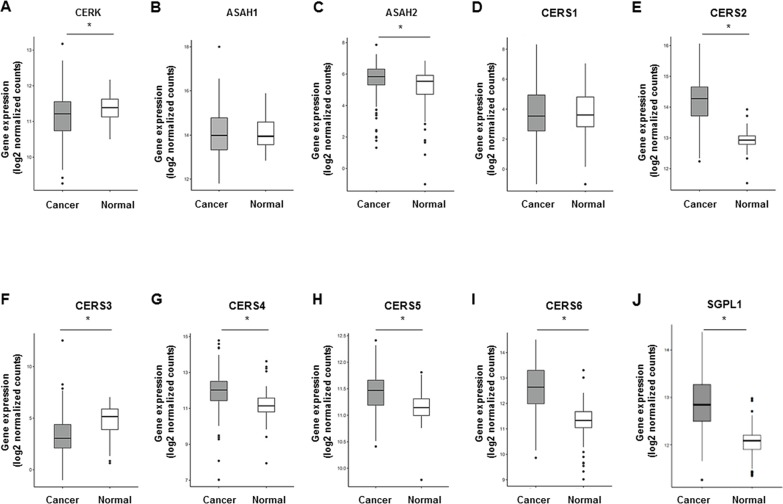
The other sphingolipid metabolic pathways and correlation between ceramide levels in breast cancer tissue and cell proliferation potency **(A-J)** The expression of genes related to the other sphingolipid metabolic pathways in The Cancer Genome Atlas (TCGA) cohort (n=112) were analyzed. Mean values are shown by the horizontal lines. ^*^, *P*<0.05 for cancer vs. normal tissue.

Ceramide 1-phosphate (C1P) is generated through direct phosphorylation of ceramide by ceramide kinase (CERK) [20]. It acts as an intracellular second messenger to promote cell survival as well as an extracellular receptor agonist to stimulate cell migration [25]. In this study, we decided not to measure the C1P levels, but rather analyzed the gene expression of CERK. This is because C1P has been shown to be too unstable to obtain any reliable measurements by our technique [26]. We found that the gene expression of CERK was significantly suppressed in cancer (Figure 6A). The gene expression of N-acylsphingosine amidohydrolase (ASAH) catalyzes the hydrolysis of the N-acyl linkage of ceramide to generate sphingosine [27] (Figure 6B, 6C). The gene expression of ASAH2 was increased in cancer (Figure 6C). At the same time, the gene expression of ceramide synthases (CS), including CS2, CS4, CS5, and CS6, which generate ceramide from sphingosine [28], were significantly increased (Figure 6D–6I). The gene expression of sphingosine-1-phosphate lyase 1 (SGPL1) which executes the final decisive step of the sphingolipid breakdown pathway, mediating the irreversible cleavage of the lipid-signaling molecule S1P [29], was also significantly increased in cancer (Figure 6J). In summary, we found that substrates to generate ceramide, including monohexocylceramide in the salvage pathway, sphingomyelin in the sphingomyelin pathway, and dihydroceramide in the *de novo* pathway were all significantly increased in cancer. Further, we found that gene expression of the enzymes that generate ceramide (GBA in the salvage pathway, SMPD in the sphingomyelin pathway, DES1 in the *de novo* pathway, and ceramide synthases in the catabolic pathway) were significantly increased in breast cancer.

### High ceramide levels are associated with low proliferation potency in breast cancer

**Figure 7:**
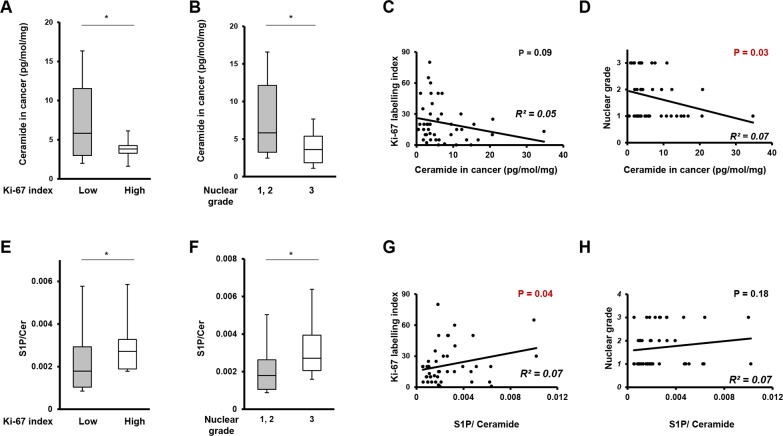
The correlation between ratio of ceramide, the ratio of ceramide to S1P or sphingosine and cell proliferation potency **(A)** The comparison of ceramide levels in breast cancer tissue between patients with Ki-67 index. Mean values are shown by the horizontal lines. ^*^, P<0.05 for high Ki-67 index vs. low Ki-67 index. **(B)** The comparison of ceramide levels in breast cancer tissue between patients with high nuclear grade (Nuclear Grade 3) and patients with low nuclear grade (Nuclear Grade 1 and 2). Mean values are shown by the horizontal lines. ^*^, P<0.05 for high nuclear grade vs. low nuclear grade. **(C)** The correlation between ceramide levels in breast cancer tissue and Ki-67 index. The correlation between two variables is denoted by R^2^. **(D)** The correlation between ceramide levels in breast cancer tissue and nuclear grade. The correlation between two variables is denoted by R^2^. **(E)** The comparison of the ratio of S1P to ceramide in breast cancer tissue between patients with high Ki-67 index and patients with low Ki-67 index. Mean values are shown by the horizontal lines. ^*^, P<0.05 for high Ki-67 index vs. low Ki-67 index. **(F)** The comparison of the ratio of S1P to ceramide in breast cancer tissue between patients with high nuclear grade (Nuclear Grade 3) and patients with low nuclear grade (Nuclear Grade 1 and 2). Mean values are shown by the horizontal lines. ^*^, P<0.05 for high nuclear grade vs. low nuclear grade. **(G)** The correlation between the ratio of S1P to ceramide in breast cancer tissue and Ki-67 index. The correlation between two variables is denoted by R^2^. **(H)** The correlation between the ratio of S1P to ceramide in breast cancer tissue and nuclear grade. The correlation between two variables is denoted by R^2^.

To determine the role of ceramide in breast cancer aggressiveness, we next investigated the relationship between the levels of total ceramide in breast cancer tissue and cell proliferation potency, using Ki-67 index and nuclear grade. Patients with high Ki67 labelling index (>30) showed significantly lower ceramide levels in cancer tissue than those with low Ki-67 index (≤30) (*p*=0.04) (Figure 7A). In investigating the association between ceramide levels and Ki-67 index within each individual patient, ceramide in cancer tissue showed a trend toward a negative association (R^2^=0.05) with levels of Ki-67 index (*p*=0.09) (Figure 7C). Regarding nuclear grade, patients with high nuclear grade were significantly associated with low ceramide levels in cancer tissue (*p*=0.04) as shown in Figure 7B. For an individual patient, ceramide levels in cancer tissue showed a significant negative association with nuclear grade (*p*=0.04) (Figure 7D).

### The ratio of S1P to ceramide is associated with proliferation potency in breast cancer

We next investigated the relationship between the ratio of S1P to ceramide levels in breast cancer tissue and cell proliferation potency. The Ki-67 index and nuclear grade were used as measures of cell proliferation. Patients with high Ki-67 (>30) had a significantly higher ratio of S1P to ceramide in cancer tissue than those with a low Ki-67 index (≤30) (p=0.03) (Figure 7E). In investigating the association between the ratio of S1P to ceramide and Ki-67 index within each individual patient, the high ratio of S1P to ceramide was significantly associated with the high levels of Ki-67 index (p=0.04) (Figure 7G). Regarding nuclear grade, high nuclear grade was significantly associated with a high ratio of S1P to ceramide in cancer tissue (p=0.04) as shown in Figure 7F. For individual patients, the ratio of S1P to ceramide showed a trend toward a positive association (R^2^=0.07) with levels of nuclear grade (p=0.18) (Figure 7H).

### Prognostic analysis for gene expression levels of ceramide-related enzymes

**Figure 8:**
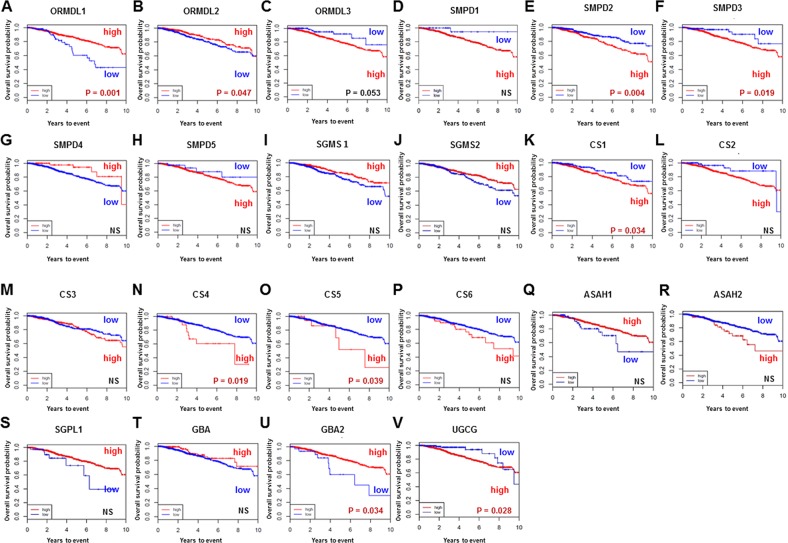
Overall survival of breast cancer patients in The Cancer Genome Atlas (TCGA) cohort The gene expression levels of enzymes related to ceramide metabolism in breast cancer tissue were examined from TCGA cohort **(A-V)**. High, patients with high gene expression. Low, patients with low gene expression. Genes are listed at the top of each figure.

We investigated overall survival and its association with high vs low gene expression level of each ceramide-related enzyme involved in each of the three ceramide-production pathways in breast cancer using TCGA cohort. First, we investigated survival significance of ORMDL (Figure 8A-8C). ORMDL, a group of genes of the Orm family, acts as a key homeostatic regulator of sphingolipid metabolism [30], and decreases ceramide levels through inhibition of serine palmitoyltransferase [31]. In this study, breast cancer patients with high expression levels of ORMDL1 and ORMDL2 showed significantly better prognosis than those with low expression levels (Figure 8A, 8B). Second, we evaluated the association of survival with each gene encoding ceramide-related enzymes in the three major pathways of ceramide synthesis. Regarding the sphingomyelin pathway (Figure 8D–8J), the patients with high levels of SMPD (SMPD2 and SMPD3) showed significantly worse prognosis (Figure 8E, 8F). Regarding the catabolic pathway (Figure 8K–8S), the patients with high expression levels of CS (CS1, 4 and 5) had significantly worse prognosis (Figure 8K, 8N, 8O), as did patients with high expression of GBA and UGCG in the salvage pathway (Figure 8T–8V). Taken together, the survival analyses of gene expression suggested that the ceramide-related enzymes that generate ceramide have an association with worse survival in breast cancer patients.

## DISCUSSION

This is the first study to demonstrate the clinical relevance of ceramide levels in breast cancer patients. The dynamic balance between S1P and sphingosine/ceramide is referred to as the “sphingolipid rheostat” and influences cancer cell fate [11]. Several studies have reported that ceramide is intimately involved in cancer pathogenesis. Alterations in its metabolism are involved in regulation of cancer initiation, progression, and/or response to chemotherapeutic agents and radiation [16, 32, 33]. However, the clinical relevance of ceramide levels in patients with breast cancer remains unclear, due to lack of accurate measurement of ceramides in human patients. In this current study, we used human breast cancer tissue and demonstrated that human breast cancer and not the peri-tumor or normal breast tissue has relatively high ceramide expression. Moreover, both substrates and enzymes of all three ceramide-biosynthesis pathways were elevated in cancer tissue, and ceramide levels were inversely associated with aggressive phenotypes of breast cancer.

Schiffmann *et al.* previously reported that the levels of ceramide in breast cancer tissue were higher than that of normal breast tissue [34]. However, the cohort of the normal tissue was small. Their sample collection period from 2001 to 2007 may have affected the sample quality through degradation over time. In contrast, we have a well-established methodology to collect and store the samples, and performed immediate measurements and analysis to avoid any deterioration of the samples [4]. Moreover, as for our cohort, we utilized a patient sample of 44 consecutive breast cancer patients and collected breast cancer tissue and normal breast tissue from the same individual patients from April, 2015 to February, 2016. To date, our study represents the largest cohort of breast cancer patients used to investigate the levels of ceramide in human breast cancer.

Unlike our previous reports where levels of S1P, sphingomyelin, and monohexocylceramide in breast cancer tissue were associated with levels in normal breast tissue among individual patients [4, 12], in this study we found that the ceramide levels of breast cancer tissue were not associated with those of normal breast tissue. In our previous study, we reported that the production of ceramide occurs independently from the condition of the host because apoptotic cells are considered to be a major source of ceramides in the cancer tissue [4]. In the present study, we clearly demonstrated that ceramide levels were increased to the highest levels within cancer and IF and to lower levels within the surrounding normal breast tissue, serum, or plasma. Considering that ceramide inhibits the expression of matrix metalloproteinases (MMPs), which are proteolytic enzymes that play a critical role of tumor invasion and metastasis [35], ceramides may suppress cancer progression without being secreted into the systemic circulation. In contrast to S1P, which acts as mediator inside and outside of cancer cells to promote their progression, ceramides may act differently in breast cancer tissue.

We also investigated the potential pathways that could be associated with increased ceramide expression in breast cancer tissue. Our data, using measurement of each sphingolipid substrate and enzyme gene expression analysis, demonstrated that all three ceramide-biosynthesis pathways are activated to increase ceramides in human cancer. It is reasonable to hypothesize that increased expression of ceramide biosynthetic enzyme levels does not necessarily imply that there is an increase in enzymatic activity since many enzymes are regulated by posttranslational modifications. Considering that both the expression of ceramide biosynthetic enzyme levels and the levels of ceramide were higher in breast cancer tissue than in normal tissue, it logically follows that the enzymatic activity would be higher in breast cancer tissue.

In the present study, we found that Ki-67 and nuclear grade were negatively associated or had a trend toward negative association with the ceramide level in cancer tissue. The high levels of ceramide in breast cancer tissue were significantly associated with low cell proliferation potency. There have been reports demonstrating the role of ceramide on cancer cell biology *in vivo* and *in vitro* [36, 37]. It has been shown that ceramide increases apoptosis in a dose dependent manner in human colon carcinoma cells [38] and in MCF-7 breast cancer cells [39]. Inhibition of the catabolic enzyme UGCG inhibited proliferation and induced apoptosis of breast cancer cells and enhanced the inhibitory effects of chemotherapeutic drugs *in vivo* and *in vitro* [40]. It has also been shown that blocking ceramide synthase decreases apoptosis in a xenograft model of human head and neck squamous cell carcinomas [41]. Moreover, models of metastases, ceramide sensitized metastatic cancer cells to apoptosis. Consistent with apoptosis sensitization activity, subtoxic doses of ceramide suppressed the metastatic potential of colon cancer cells in an experimental lung metastasis mouse model, as well as breast cancer growth and spontaneous lung metastasis in a breast cancer mouse model [38]. Taken together, these *in vivo* and *in vitro* studies indicate that ceramide increases apoptosis in a dose dependent manner, regardless of the cancer cell origin. These *in vivo* and *in vitro* studies support our clinical results, which revealed that high ceramide levels in breast cancer were associated with less aggressiveness of cancer.

We also performed a survival analysis based on the gene expression encoding each ceramide-related enzyme to clarify the prognostic impact of the activation status of the three ceramide-biosynthetic pathways. Interestingly, the prognostic evaluation suggested that the ceramide-related enzymes related to increased ceramide expression were associated with worse survival in breast cancer patients. Given that ceramide is one of the components of the cellular membrane [42], cancer cells with a high proliferation rate would be expected to require more ceramide. Ceramide also plays a role in apoptosis in cancer tissue. In the context of the “sphingolipid rheostat” that converts ceramide produced in cancer tissue into S1P, it is reasonable to hypothesize that cancer cells may become more aggressive from high levels of S1P converted from ceramide. Indeed, we found that a high ratio of S1P to ceramide was associated with high proliferation potency, reflected to the high Ki-67 index and nuclear grade. Therefore, it appears that the worse prognosis of patients with high expression of ceramide-related enzymes was due to S1P converted from ceramide by the “sphingolipid rheostat”.

We also found that breast cancer patients with low expression levels of ORMDL1 and ORMDL2 had a significantly worse prognosis than those with high expression levels. Recently, ORMDLs have emerged as key proteins for maintaining sphingolipid homeostasis [30]. ORMDLs act as negative regulators of sphingolipid synthesis that form a conserved complex with serine palmitoyltransferase, the first and rate-limiting enzyme in sphingolipid production. ORMDLs may also “sense” ceramide and decrease its synthesis through negative feedback mechanisms [43–45]. Our findings suggest that the low ORMDL expression may result in dysregulation of sphingolipid homeostasis, which leads to worse prognosis for the patients. However, the precise mechanisms of this phenomenon remain undefined.

Ceramide is receiving a lot of attention in clinical research [46, 47]. Sphingolipids including ceramide have been implicated in the mechanism of action of cancer chemotherapeutics [48–50]. It has been shown that ceramide is a key regulator of the taxane-mediated spindle assembly checkpoint and taxane-induced cell death [51]. Recently, Jing et al studied the resistance to chemotherapy in advanced breast cancer by using 30 patients with stage IV breast cancer tissues. Pro-apoptotic ceramide was significantly lower in all patients after chemotherapy, suggesting that downregulation of ceramide is a common feature of breast cancer patients in response to chemotherapy. Stimulating ceramide or decreasing catabolic enzymes such as UGCG can potentially be useful for breast cancer treatment [40]. Additionally, ceramide has also been evaluated as a diagnostic biomarker. Serum levels of sphingolipid metabolites have been shown to have a significant upregulation in patients with hepatocellular carcinoma (HCC) as compared to patients with cirrhosis. Specifically, C16-ceramide levels may serve as a novel diagnostic biomarker for the identification of HCC in patients with liver diseases [52].

We recognize that there are limitations of our study. First, there is a limitation regarding the generalizability of this work, in that all patients were Japanese. Also, all patients in this study had mastectomy because it is difficult to collect the peri-tumor and normal breast tissue samples from the patients who undergo lumpectomy. Nonetheless, our study represents the largest cohort of patients used to investigate the levels of and potential role of ceramide in human breast cancer. Future studies using larger cohorts may provide additional information regarding the association between ceramide levels and subtypes of breast cancer or the association between ceramide levels and drug-resistance. The current analyses are based on homogenates of a large population of cells. Thus, this represents an important limitation in addressing sphingolipid metabolism at the individual cell level. Moreover, the mass spectrometric analysis was limited to specific fractions of sphingolipids. Other sphingolipids that are not easily determined by mass spectrometry but may play a role in carcinogenesis, such as gangliosides, were not included in this study. All the data in this study were obtained from human samples and observed results. We did not conduct any experiments to reveal correlation between ceramide metabolism and breast cancer cell biology. With that said, there has been numerous *in vivo* and *in vitro* studies that revealed correlation between ceramides and tumor biology by the others [53–55]. Our results are in agreement with the previous studies, and we reported the association between ceramides and human breast cancer utilizing clinical samples for the first time.

This is the first study to elucidate the clinical relevance of ceramide metabolism in breast cancer patients. We demonstrated that ceramide levels in breast cancer tissue are significantly higher than those in normal tissue, with activation of the three ceramide biosynthesis pathways. Further, we demonstrated that ceramide levels in breast cancer tissue are inversely associated with aggressive phenotype. Moreover, we showed the prognostic impact of the activation status of enzymes in the three ceramide synthetic pathways. Further studies are needed to reveal the underlying mechanisms of how ceramide and sphingolipid homeostasis affect oncologic processes in breast cancer patients.

## MATERIALS AND METHODS

### Patient tissue samples

This study was approved by the Institution Review Board of Niigata University Medical and Dental Hospital. Breast cancer tissue specimens were collected between April 2015 and February 2016 from 62 consecutive breast cancer patients. All patients underwent simple mastectomy in Niigata University Medical and Dental Hospital. Of the 62 patients, 18 patients received neoadjuvant treatment (chemotherapy, radiation therapy, or hormone therapy) and were excluded from this study. The remaining 44 patients constituted the final cohort. Patient characteristics included age, body mass index (BMI), pathologic T stage, pathologic N stage, histopathology, Ki-67 index, and nuclear grade. Pathology was conducted by independent pathologists in the Department of Pathology at Niigata University Medical and Dental Hospital.

Breast cancer tissue (n = 44), peri-tumor tissue (n = 36), and normal breast tissue (n = 44) were collected from surgical specimens immediately after the operation and preserved in liquid nitrogen. Peri-tumor tissue was defined as tissue within 1 cm from the gross edge of tumor. Of 44 patients, pert-tumor tissue could not be collected from 8 patients because peri-tumor tissue specimens were unable to be identified macroscopically. Normal breast tissue was obtained from a different quadrant of the breast where cancer was not located. Cancer and normal breast tissues were also collected for interstitial fluid (IF) measurement, and IF was prepared using our centrifugation technique described previously. All tissue samples were stored at -80°C.

### Quantification of sphingolipids by liquid chromatography, electrospray ionization-tandem mass spectrometry (LC-ESI-MS/MS)

We utilized our established method to measure sphingolipids within the tissue samples obtained from this patient cohort [4, 13]. Internal standards were purchased from Avanti Polar Lipids (Alabaster, AL, USA) and added to samples in 20 μl of an ethanol: methanol: water (7:2:1) cocktail of 500 pmol each. The high-performance liquid chromatography (HPLC) grade solvents were obtained from VWR (West Chester, PA, USA). Lipids were extracted from tissue, and sphingolipids were quantified by LC-ESIMS/MS (4000 QTRAP, ABI) as described previously by our group [56, 57].

### Gene expression analysis of ceramide-producing enzymes using the cancer genome atlas (TCGA) cohort

To evaluate the expression level of each gene encoding enzymes involved in the three ceramide production pathways (i.e. salvage pathway, sphingomyelin pathway, and the *de novo* pathway), the RNA-sequence gene expression quantification data of breast cancer tissue (n = 112) and paired normal breast tissue (n = 112) were retrieved from the Genomics Data Commons (GDC) data portal. The gene expression levels were derived using normalization methods provided in DESeq2 package [58].

### Prognostic analysis for gene expression levels of ceramide-related enzymes using TCGA cohort

To evaluate the prognostic impact of the gene expression level of each ceramide-related enzyme involved in the three ceramide-production pathways, the RNA-sequence gene expression quantification data of breast cancer tissue (n = 1047) were retrieved from the GDC data portal and analyzed as previously reported [18]. For survival analyses using Cox proportional hazard model based on expression of each single gene, patients were classified as having high or low expression of the given gene using a gene-specific threshold. All patients were labeled as “high” if the expression of the interrogated gene was above the threshold and “low” if it was below the threshold. To determine the gene-specific threshold, a running Cox proportional hazard statistics was applied. Patients were dichotomized based on multiple candidate cutoff points within the range of observed gene expression values, and the optimal cutoff points were chosen based on statistical significance of the model [20].

### Statistical analysis

Patient characteristics were reported using the mean and standard deviation for continuous variables, and using frequencies and relative frequencies for categorical variables. The Mann-Whitney U-test and Student's *t*-test for unpaired data were performed to compare differences between breast cancer tissue and normal tissue. The Kruskal Wallis H test for unpaired data was performed to compare differences among breast cancer tissue, peri-tumor tissue, and normal breast tissue. Receiver-operating characteristic (ROC) curves and the area under the ROC curve (AUC) were used to compare ceramide levels between cancer tissue and normal breast tissue as well as cancer tissue and peri-tumor tissue. All statistical evaluations were performed using the SPSS 16.0J software package (SPSS Japan, Tokyo, Japan). All tests were 2-sided and a *P* value of less than 0.05 was considered statistically significant.

## Abbreviations

ASAH: N-acylsphingosine amidohydrolase
AUC: the area under the ROC curve
BMI: body mass index
CS: ceramide synthase
CERK: ceramide kinase
C1P: ceramide-1-phosphate
DES-1: dihydroceramide desaturase 1
ER: estrogen receptor
GBA: glucosylceramidase
GDC: genomics data commons
HCC: hepatocellular carcinoma
IF: interstitial fluid
ROC: receiver-operating characteristics
SGMS: sphingomyelin synthase
SMPD: sphingomyelin phosphodiesterase
S1P: sphingosine-1-phosphate
SGPL1: sphingosine-1-phosphate lyase 1
TCGA: the cancer genome atlas
TNF-α: tumor necrosis factor-α
UGCG: uridine diphosphoglucose ceramide glucosyltransferase
